# Sex-Specific Properties of Astrocytes: From Development to Evolutionary Insights

**DOI:** 10.1007/s11064-025-04512-w

**Published:** 2025-08-13

**Authors:** Mariza Bortolanza, Davide Gobbo, Lipao Fang, Anja Scheller, Xianshu Bai, Frank Kirchhoff

**Affiliations:** 1 Molecular Physiology, Center for Integrative Physiology and Molecular Medicine (CIPMM), University of Saarland, 66421 Homburg, Germany; 2 Center for Gender-specific Biology and Medicine (CGBM), University of Saarland, 66421 Homburg, Germany; 3 School of Pharmacy, China Pharmaceutical University, Jiangning Campus, Longmian Avenue 639, Nanjing, 211198 China

**Keywords:** Astrocytes, Sex, Development, Aging, Cross-species

## Abstract

Exploring sex-specific differences in astrocytes has emerged as a vital area of neurobiological research. This research sheds light on how astrocytes in females and males differ in their contribution to neuronal functionality, overall brain health, and various neurological disorders. These cells play a critical role in sustaining homeostasis, providing metabolic support, facilitating neurotransmitter recycling and responding to injuries to the central nervous system. Their physiology exhibits significant variability, which is influenced by factors such as sex, developmental stage, species differences and environmental conditions. This review provides an integrated overview of these factors, addressing key themes including developmental dynamics, aging, signalling mechanisms, glial interactions, responses to pathological states and cross-species comparisons.

## Introduction

Astrocytes, which account for 20–40% of all glial cells in different regions of the central nervous system (CNS), are critical regulators of neuronal homeostasis, synaptic modulation, blood-brain barrier (BBB) maintenance, and neuro-immune responses [1–3]. Importantly, accumulating evidence indicates that astrocyte physiology is not uniform across individuals, but instead is modulated by biological sex, with significant implications for brain development, aging, and disease susceptibility [4, 5].

Sex-specific differences in astrocytic morphology, transcriptomics, and functional outputs, such as Ca^2+^ signalling and cytokine release, have been demonstrated in various regions of the CNS [6–10]. These differences are evident early in development and are influenced by both organisational effects of sex hormones and direct genetic contributions from sex chromosomes [11–14]. During aging, astrocytes exhibit sex-dependent changes in reactivity, metabolism, and gene expression that may underlie distinct susceptibility to neurodegenerative diseases such as Alzheimer’s (AD), Parkinson’s (PD) and multiple sclerosis (MS) [15, 16].

Genetic and epigenetic mechanisms, including X-chromosome inactivation patterns, hormone receptor expression, and sex-biased DNA methylation, further regulate astrocyte behaviour over their lifespan [5, 17, 18]. Under stress and injury contexts, astrocyte plasticity exhibits notable sex differences, influencing glial scar formation, cytokine secretion, and recruitment of peripheral immune cells [19–22]. Furthermore, astrocytic signaling pathways are modulated by sex hormones and genetic background, which can alter neuronal-glial interactions and circuit function [23–26].

Cross-species studies highlight both conserved and divergent aspects of astrocyte biology. While certain sex-specific patterns are preserved in rodents and humans, others appear species-specific, underscoring the need for comparative analyses to improve translational relevance [27, 28]. Integrating data across species, developmental stages, and physiological conditions will enhance our understanding of astrocyte-mediated mechanisms in brain health and disease.

This review examines how sex shapes astroglia biology from development through aging, focusing on genetic and epigenetic regulators, responses to injury, as well as intra- and intercellular signalling. We also emphasize how species differences underscore our understanding of astrocyte diversity and the need for sex-aware research in basic and translational neuroscience.

### Sex-Specific Developmental Influence on Astrocyte Functions

Astrocytes exhibit sex-specific differences in number, activity and development, affecting cognitive functions such as memory and susceptibility to neurodegenerative diseases [28, 29]. These differences are reflected in the modulation of astrocytic receptors and their downstream signalling pathways [30]. However, sex differences do not begin with the onset or progression of a neurodegenerative disease, but rather during brain development, and therefore, the basis for age- or disease-related sex differences may be laid already during neonatal and postnatal brain development. Understanding how sex hormones shape astrocytic development is fundamental for comprehending their functional roles later in life.

In cell culture studies using astrocytes derived from female rats and mice, it has been demonstrated that exposure to sex steroids significantly affects their morphology and function. Specifically, female astrocytes showed enhanced responses to estradiol, contributing to their proliferation and increasing neuroprotective capabilities against stressors such as oxidative damage, lipotoxicity and neuroinflammation [21, 31].

However, to date, there is no comprehensive understanding of the sex differences in astroglial development (including astrogenesis, neurogenesis, synaptogenesis and synapse pruning) in mice and humans. A study in adult rats suggests that sex differences in the number and complexity of adult astrocytes may be influenced by testosterone, and that estradiol may also be the basis for developmental differences. Males consistently have more astrocytes than females in the amygdala and hypothalamus until adulthood, while the opposite is true in the hippocampus, indicating interesting developmental differences between brain regions [29, 32, 33]. The number of astrocytes was increased in both sexes after testosterone treatment, but the difference was more pronounced in males [29]. Studies showed that astrocytes partially express estrogen, androgen, and progesterone receptors in a sex-specific manner, making them susceptible to direct interference [29, 34]. In addition, recent comprehensive data based on meta-analysis in both sexes show brain-specific differences, but not yet at the cellular level, especially in astrocytes [35]. Transcriptomic analysis of neocortical astrocytes revealed two distinct phenotypes important for glio- and synaptogenesis in early development. Sex-specific differences in gene expression patterns during development peaked at P7 and P14 and appeared to be due to males reaching a mature astroglial phenotype earlier than females. The earlier maturation in males could be related to the influence of sex hormones, particularly estrogen, which is known to influence brain development. The perinatal testosterone surge, after aromatisation (conversion) to estradiol, leads to altered gene expression and subsequently to faster maturation of astroglia in males. Important targets of these changes are the astroglially expressed gonadal hormone receptors (estrogen receptor alpha (ERα), ER beta (Erβ), G protein-coupled ER), allowing a rapid astroglial response to circulating estrogens. The expression of astroglial markers such as glial fibrillary acidic protein (GFAP) and glutamine synthetase is not affected by perinatal estrogen, whereas astroglial maturation markers such as vimentin, aldehyde dehydrogenase 1 family, member A1 (Aldh1a1) and iodothyronine deiodinase 2 (Dio2) are. These developmental differences between the sexes may influence the assembly of neuronal networks and represent interfaces that are susceptible to disruption and disease [36, 37].

Finally, all the studies that analysed astroglial sex differences have examined women either at an undisclosed time or at a specific time in their menstrual cycle. For example, Rurak et al. [36] evaluated women in the metoestrus phase, when ovarian hormones are at their lowest and therefore closer to the levels typically observed in men. However, sex differences at the transcriptomic level may be more pronounced during the proestrus phase, when estrogen levels peak. Further research is therefore needed to clarify the sex differences in astroglia.

### Sex-Dependent Changes in Astrocytic Responses with Aging

Astrocyte influences on brain physiology are important not only during development but also throughout aging. With age, astrocytes become increasingly reactive, often adopting a pro-inflammatory phenotype. This shift is reflected by elevated levels of reactivity markers such as GFAP, which increase particularly in females. Aged female mice exhibit widespread GFAP upregulation across multiple brain regions, indicating global astrocytic activation [38]. While male astrocytes also undergo astrogliosis, some astrocytic functions, such as glutamate uptake, tend to decline more markedly with age in males [3, 39].

**Fig. 1:**
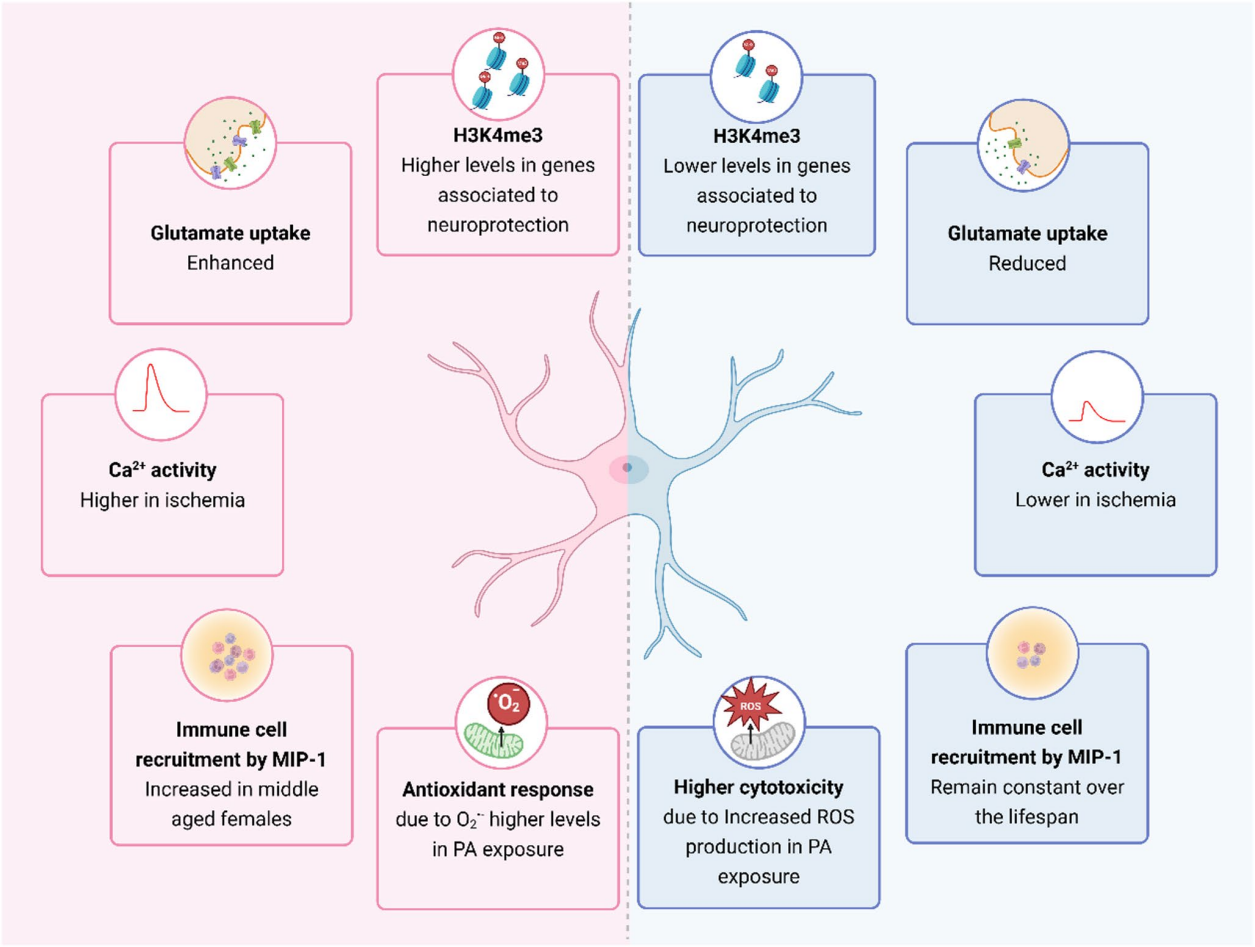
Sex-dependent functional and regulatory differences detected in astrocytes. Sex differences in astrocyte function, between females (left, pink) and males (right, blue). H3K4me3, H3-trimethyl lysine-4; MIP-1, Monocyte chemoattractant protein-1; PA, palmitc acid; ROS, reactive oxygen species. Created in https://BioRender.com

This sex-specific pattern of astrocyte aging may be partly due to hormonal differences. In postmenopausal women, the decline in estrogen correlates with increased levels of pro-inflammatory cytokines such as interleukin-6 (IL-6) and tumour necrosis factor-alpha (TNFα). This, in turn, may contribute to the elevated neuroinflammation and increased risk of dementia in women [40]. Animal studies support this pattern. For example, aged female rats subjected to focal ischemia exhibit higher levels of cytokines and chemokines than younger females, indicating enhanced astrocytic activation [41]. These astrocytes also secrete higher levels of macrophage inflammatory protein-1 (MIP-1), suggesting an increased ability to recruit immune cells, and reduced production of Insulin-like Growth Factor 1 (IGF-1), a trophic factor known to promote neuroprotection and improve glutamate transporter activity [41](for review [39]). In contrast, male astrocytes maintain similar functional capabilities across age [41] (Fig. 1). These sex-based differences in astrocytic responses may affect disease progression after brain injury. Clinically, women at postmenopausal age tend to experience worse outcomes after stroke than age-matched men, including larger infarcts and poorer recovery [42–45]. Similarly, middle-aged female rats display more extensive brain damage following ischemia compared to both younger females and age-matched males [41, 46].

Age-related changes in astrocytic plasticity are further complicated by environmental stressors. For example, chronic restraint stress induces astrocytic hypertrophy in the female prefrontal cortex, a response modulated by ovarian hormones. By contrast, male astrocytes, which are initially larger, may undergo atrophy not only in response to stress but also as a consequence of aging [47–50]. Together, these findings suggest that the hormonal shifts associated with aging drive female astrocytes toward a more reactive and less neuroprotective state [44], reinforcing the need for sex-specific approaches in the treatment of age-related brain disorders.

### Genetic and Epigenetic Modulators of Sex-Specific Astrocyte Aging

Beyond hormonal influences, genetic risk factors contribute to sex-dependent astrocytic vulnerability. Among the three major human alleles of the apolipoprotein E (APOE), the APOE4 isoform strongly associated with age-related neurodegenerative diseases, has been shown to exacerbate astrocytic inflammation. Astrocytes expressing the APOE4 allele, particularly in the female population, exhibit increased baseline inflammation and express 1.5–2.5 times more IL-6, interleukin-1 beta (IL-1β) and TNFα than their male counterparts, therefore highlighting a sex-specific vulnerability to inflammation associated with the APOE4 genotype [51].

This interplay of sex and genetic background plays a pivotal role in determining individual susceptibility to neurodegenerative disease. In postmenopausal women, particularly those carrying the APOE4 allele, astrocyte-driven inflammation may exacerbate AD pathology. Early in life, women typically exhibit more effective astrocyte-mediated vascular support and tissue repair after stroke [52], but these benefits decline after menopause, contributing to poorer outcomes [44, 52]. Therapeutic strategies that enhance IGF-1 secretion or glutamate clearance have been promising in improving recovery in aged female animal models [44]. In males, preservation of key metabolic functions in astrocytes, including glutamate transport and mitochondrial health, may be more critical for neuroprotection [39]. Overall, targeting sex-specific aspects of astrocytic aging holds the potential for more effective interventions in neurodegenerative disease and recovery from brain injury.

Sex chromosomes and epigenetic regulation also contribute to the divergent aging trajectories in astrocytes. Genes on the female X-chromosome can escape inactivation or undergo age-dependent expression shifts [53]. Transcriptomic analysis of human astrocytes reveals sex-specific differences in gene expression, including chromatin remodelling factors [27, 54]. Epigenetic control also differs during aging: compared to middle-aged females, younger female astrocytes show higher levels of the activating histone H3-trimethyl lysine-4 (H3K4me3) mark, which is known to promote gene activation. This increase is observed at gene *loci* associated with neuroprotection, such as the vascular endothelial growth factor (VEGF) and the micro ribonucleic acid-17 ~ 92 (miR-17 ~ 92) [44, 55], supporting increased expression of VEGF and miR-20a after stroke. This decline in epigenetic support with age diminishes astrocyte-mediated neuronal protection in females. In males, other epigenetic mechanisms - possibly involving androgen-regulated genes - may be involved but are still less understood.

### Sex Differences in Astrocyte Aging Across Species

Investigating astrocytic function across species provides valuable insights into the evolutionary mechanisms governing these glial cells. While foundational research has heavily relied on rodent models such as C57BL/6 mice and Sprague-Dawley rats, studies on astrocytes in higher primates, particularly their sex-specific roles, remain limited. Emerging evidence from human studies highlights significant species-specific variations in astrocyte function and regulatory mechanisms. Human astrocytes exhibit greater morphological complexity than their rodent counterparts [47], featuring more extensive branching and a higher density of processes, which may enhance their communication with neurons. Additionally, specialised astrocyte populations, such as interlaminar astrocytes in primates, have been identified in humans and higher primates but are absent in other species [28, 56] (reviewed in [57]). This unique astrocyte population likely contributes to the more complex neural networks and may result in different aging responses. In line with this, growing evidence indicates that rodents typically show earlier and more extended GFAP upregulation throughout the brain with age [38], whereas primates show features of cellular senescence, including increased p16^INK4a^ expression in cortical astrocytes, a marker associated with neurodegeneration [58]. Spinal cord studies reveal that some age-related changes in astrocyte markers displaying sex dimorphisms differ across species [27]. For example, GFAP increases with age in female primates (e.g. mouse lemurs and humans), but not consistently in rodents [27]. These species-dependent features highlight the importance of primate models for translational aging research.

### Sex-Specific Regulation of Astrocytic Signalling Pathways and Ca^2+^ Dynamics

The response to different neurotransmitters also shows sex differences between male and female astrocytes. Most strikingly, in vitro and in vivo rat models have revealed sex-dimorphic responses to glutamate for male and female astrocytes [59, 60] as well as differences in glutamate uptake [25]. In particular, estrogen exposure results in the upregulation of the excitatory amino acid transporter 2 (EAAT2) via nuclear factor-kappa B (NF-κB) and cyclic AMP-responsive element-binding protein (CREB) signaling pathways and enhances the expression of both excitatory amino acid transporter 1 and 2 (EAAT1 and EAAT2) through a transforming growth factor-alpha (TGF-α) mediated pathway [61]. This suggests that male and female astrocytes may differ in their capacity to remove excess glutamate from the synaptic cleft, thereby reducing excitotoxicity. In line with this, studies from ovariectomized adult female rats, whose estrogen levels drop upon surgical removal of the ovaries, revealed decreased sodium–potassium adenosine triphosphatase (Na⁺/K⁺-ATPase) activity [62]. Since the activity of the Na⁺/K⁺-ATPase is essential for correct ionic homeostasis and glutamate transport [63], the exposure to estrogens could cause female astrocytes to become more efficient in glutamate uptake and maintaining low extracellular glutamate levels.

More recently, changes in the expression of the astrocytic metabotropic glutamate receptor 3 (mGluR3), which is particularly abundant in the hippocampal and cortical astrocytes, have been shown to affect spatial memory performance in a sex-dependent manner in mice [30]. Specifically, reduced mGluR3 levels impaired spatial memory in female but improved it in male mice, while increases in mGluR3 expression enhanced memory in females. Moreover, acute chemogenetic stimulation of G_i/o_-coupled or G_s_-coupled receptors in the hippocampal astrocytes resulted in bidirectional and sex-dimorphic effects, suggesting that the astrocytic regulation of memory involves a sex-specific balance between G_s_-coupled and G_i/o_-coupled receptor signalling. These findings raise the possibility that G protein-coupled receptor signalling in astrocytes may contribute to sex-specific differences in the pathophysiology of the CNS.

Estrogens, and in particular estradiol, can stimulate astrocytes to produce growth factors such as brain-derived neurotrophic factor (BDNF) and IGF-1 [17], making astrocytes key regulators of neuronal survival and growth, as well as neurotransmitter modulation and neuronal plasticity. In addition, it has been reported that estrogens modulate astrocytic Ca^2+^ signalling, which is a key component of astrocytic signalling and activation stage and is crucial for gliotransmitter release [64]. This implies that astrocytic Ca^2+^ activity and subsequent gliotransmission, which in turn affect neuronal survival and the modulation of neural networks, may be under the control of gonadal hormones. Indeed, astrocytes, as key components of the BBB, which sex hormones can easily cross due to their lipophilic nature and small size, likely represent crucial targets of circulating gonadal hormones. As a result, sex hormones may represent key modulators of astrocytic Ca^2+^ responses under pathophysiological conditions affecting the CNS [23].

Next to gliotransmission, the astrocytic metabolism, as well as the metabolic coupling between astrocytes and neurons, is coordinated by transient intercellular Ca^2+^ waves [24]. Thus, the existence of sex differences in astrocytic Ca^2+^ signalling may be highly relevant in the context of conditions, such as brain injury, in which the astrocytic metabolism is essential to maintain neuronal function in the damaged tissue. For instance, following middle cerebral artery occlusion in adult mice, astrocytes in the ipsilateral hemisphere of female mice display more frequent Ca^2+^ elevations compared to males [65], possibly contributing to the sex differences observed in the incidence and severity of stroke events in humans. Similarly, in neonatal hypoxia-ischemia, female astrocytes exhibit enhanced mitochondrial metabolism compared to males [66], a metabolic advantage that may contribute to sex differences in neuronal vulnerability, as neuronal metabolic activity is closely dependent on astrocyte metabolic support [66, 67]. In addition, male and female astrocytes in the hypothalamus respond differently to systemic metabolic challenges and show different gliosis levels as well as inflammatory response [68].

In parallel to that, several studies have reported sex-dimorphic gene expression in astrocytes following systemic inflammation [19–21], oxidative stress [69, 70], as well as in vivo cortical brain injuries [22]. Estradiol also regulates astrocytic proliferation, which increases following brain injury to form the glial scar and restore tissue homeostasis [71]. Studies in primary mouse cortical astrocytes have shown that the regulation of astrocyte proliferation by estradiol is different in male and female astrocytes and depends on the activation of the extracellular signal-regulated kinases (ERK) signalling in female, but not in male [72, 73].

### Sex Differences in Regulation of Reactive Astrogliosis and Immune Cell Recruitment After Injury

**Fig. 2:**
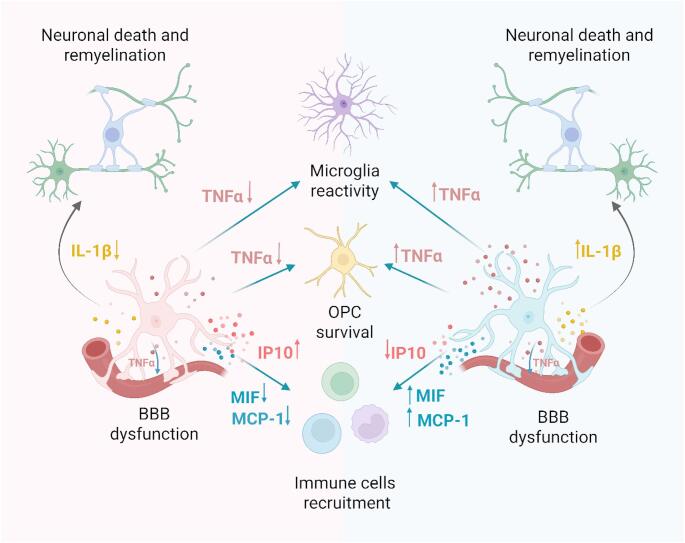
Sex-specific astrocyte-mediated crosstalk in neuroinflammation and remyelination

Sexual dimorphism significantly influences how glial cells respond to CNS injury, particularly in shaping astrocytic reactivity [74]. These variations in astrocyte behaviour may, in turn, alter their communication with neurons, microglia, oligodendrocytes and infiltrating immune cells. For example, male mice exhibit a more pronounced and sustained inflammatory response during the chronic phase of stroke, characterized by a higher infiltration of T cells [75]. Consistently, Acaz-Fonseca et al. [22] reported in males a higher percentage of astrocytes expressing monocyte chemoattractant protein-1 (MCP-1), also referred to as chemokine (C-C motif) ligand 2 (CCL2), a chemokine involved in the recruitment of immune cells and the regulation of gliosis (Fig. 2). This correlates with a higher density of ionized calcium–binding adaptor molecule 1 (IBA1)-immunoreactive cells in the area close to the lesion.

Similar sex-dependent patterns in astrocytic behaviour have been observed in chronic neuroinflammatory conditions such as MS. Elevated levels of macrophage inhibitory factor (MIF, Fig. 2), a proinflammatory cytokine involved in astrocyte-mediated responses to injury and inflammation, have been detected in the serum of male patients with progressive MS lesions [76, 77]. In contrast, Hjaresen et al. [78] reported lower MIF levels in female patients during the early phase of the disease and did not observe significant sex differences during the progressive stage. Notably, MIF levels positively correlate with disease severity and the extent of neuronal loss [79]. Supporting these clinical observations, female mice with experimental autoimmune encephalomyelitis (EAE) exhibit lower levels of MIF [80].

Beyond autoimmune diseases, a stronger inflammatory response was observed in male mice after lipopolysaccharide (LPS) stimulation. For example, primary astrocytes derived from male neonatal mice exhibit a more pronounced increase in IL-6, TNFα, and IL-1β levels upon exposure to LPS compared to female astrocytes, which exhibit increased interferon-gamma induced protein 10 kD (IP10, Fig. 2) [21]. The authors suggest that perinatal exposure to testosterone may prime male astrocytes to respond differently to inflammatory stimuli [21]. Additionally, sex hormones such as estrogen and progesterone have been shown to reduce LPS-induced TNFα and IL-18 expression in midbrain astrocytes, indicating that sex steroids modulate neuroinflammatory processes and may exert protective effects in female astrocytes [81].

Astrocytes from males (right, blue) and females (left, pink) respond differently to inflammatory stimuli, which in turn influence surrounding cells. In both sexes, TNFα produced by astrocytes promotes reactive microgliosis, decreases OPC survival, and contributes to BBB dysfunction. IL-1β release exacerbates neuronal injury and impairs remyelination. Additionally, astrocyte-derived chemokines and cytokines such as IP-10, MIF, and MCP-1 modulate the recruitment of peripheral immune cells. These sex-dependent astrocyte interactions shape the local inflammatory environment, ultimately influencing remyelination efficiency and the extent of neuronal damage. BBB, blood-brain barrier; IL-1β, Interleukin-1 beta; IP10, interferon-gamma induced protein 10 Kd; MCP-1, monocyte chemoattractant protein-1; MIF, macrophage inhibitory factor; OPC, oligodendrocyte progenitor cell; blood-brain barrier (BBB). Created in https://BioRender.com.

Astrocytes also contribute to the sexually dimorphic response to metabolic challenges [82]. For instance, in male rats, neonatal overnutrition leads to increased astrocyte number and elevated TNFα levels in late adulthood, which are associated with overweight and a greater susceptibility to obesity-related pathologies [68]. In parallel to that, male and female astrocytes respond differently to specific metabolic insults such as palmitic acid (PA), which is a key component of high-fat diets known to affect brain metabolism. Exposure to PA slows down the Krebs cycle and the glutamate-glutamine cycle while inducing higher levels of IL-6, IL-1β and GFAP in male hypothalamic astrocytes [83, 84]. Moreover, PA triggers greater cytotoxicity in male cortical astrocytes associated with an increase in reactive oxygen species (ROS) production(Fig. 1), which might promote a state of chronic inflammation [34]. Conversely, female astrocytes showed an antioxidant response characterised by elevated mitochondrial superoxide ion (O2^⋅‒^) levels (Fig. 1) and upregulation of proteins such as catalase, glutathione peroxidase-1 (Gpx-1) and nuclear factor erythroid 2-related factor 2 (Nrf2), providing protective signals to surrounding cells [34]. Building on this, other evidence suggests that sex hormones such as estradiol exert protection against PA-induced damage. In males, estradiol reduces the activation of inflammatory markers, including phosphorylated c-Jun N-terminal kinase (pJNK), TNFα and caspase-3, whereas in females, it decreases apoptotic cell death without affecting these inflammatory pathways [31]. Supporting this, deletion of ERα abolishes the anti-inflammatory effects of estradiol, leading to increased PA-induced inflammation, thereby highlighting the essential role of ERα in mediating these sex-specific protective mechanisms [84].

Further evidence of sex-specific astrocytic responses has emerged from studies on short-chain fatty acids deriving from gut microbiota. Spichak et al. [85] showed that butyrate increases *Bdnf* and Peroxisome proliferator-activated receptor gamma coactivator 1-alpha *(Pgc1-α*) expression in females. In contrast, acetate specifically increases aryl-hydrocarbon receptor (*Ahr*) and *Gfap* expression in males, while propionate is linked to increased IL-22 in males but not in females. These findings suggest that microbial metabolites can modulate astrocytic gene expression in a sex-dependent manner.

In summary, astrocytic responses to injury, inflammation, and metabolic stress are strongly influenced by sex, with implications for disease susceptibility, progression, and therapeutic strategies. These differences are mediated by both intrinsic programming and hormonal modulation, underlining the need to incorporate sex as a biological variable in studies of neuroinflammation and glial biology.

## Conclusion

In conclusion, the investigation of sex-specific differences in astrocytes provides a complex and multifaceted perspective that carries substantial implications for the field of neurological sciences. Factors including developmental variations, age-related changes, signalling mechanisms, intercellular interactions, and species differences collectively portray astrocytes as integral players in both health and disease.

Recognising sex as a pivotal variable is essential for better understanding how astrocytes influence neurological outcomes. As research continues to evolve, it will be crucial to adopt a holistic approach that incorporates sex, species variability, and environmental factors. Such comprehensive methodologies will ultimately enhance our understanding of glial biology, leading to therapeutic strategies that are better tailored to address the distinct needs observed across diverse individuals and conditions. The works reviewed herein lay the groundwork for future evaluation of the critical effects of sex on astrocytic functions and their implications for brain health and disease.

## Data Availability

No datasets were generated or analysed during the current study.

## Abbreviations

AD: Alzheimer’s disease
Ahr: Aryl-hydrocarbon receptor
Aldh1a1: Aldehyde dehydrogenase 1 family, member A1
APOE4: Apolipoprotein E4
BBB: Blood-brain barrier
BDNF: Brain-derived neurotrophic factor
CNS: Central nervous system
CREB: Cyclic AMP-responsive element-binding protein
Dio2: Iodothyronine deiodinase 2
EAAT1: Excitatory Amino Acid Transporter 1
EAAT2: Excitatory Amino Acid Transporter 2
EAE: Experimental autoimmune encephalomyelitis
ERK: Extracellular signal-regulated kinases
ERα: Estrogen receptor alpha
ERβ: Estrogen receptor beta
GFAP: Glial fibrillary acidic protein
Gpx-1: Glutathione peroxidase-1
H3K4me3: H3-trimethyl lysine-4
IBA-1: Ionized calcium–binding adaptor molecule 1
IGF-1: Insulin-like growth factor 1
IL-1β: Interleukin-1 beta
IL-6: Interleukin-6
IP10: Interferon-gamma induced protein 10 kD
iPSCs: Induced pluripotent stem cells
LPS: Lipopolysaccharide
MCP-1/CCL2: Monocyte chemoattractant protein-1/ chemokine (C-C motif) ligand 2
mGluR3: Metabotropic glutamate receptor 3
MIF: Macrophage inhibitory factor
MIP-1: Macrophage inflammatory protein-1
miR: MicroRNA
mRNA: Messenger ribonucleic acid
MS: Multiple sclerosis
Na⁺/K⁺-ATPase: Sodium–potassium adenosine triphosphatase
NF-κB: Nuclear factor-kappa B
Nrf2: Nuclear factor erythroid 2-related factor 2
PA: Palmitic acid
PD: Parkinson´s disease
Pgc1-α: Peroxisome proliferator-activated receptor gamma coactivator 1-alpha
pJNK: Phosphorylated c-Jun N-terminal kinase
ROS: Reactive oxygen species
TGF-α: Transforming growth factor-alpha
TNFα: Tumour necrosis factor-alpha
VEGF: Vascular endothelial growth factor
